# Crystal Structure of Phototoxic Orange Fluorescent Proteins with a Tryptophan-Based Chromophore

**DOI:** 10.1371/journal.pone.0145740

**Published:** 2015-12-23

**Authors:** Nadya V. Pletneva, Vladimir Z. Pletnev, Karen S. Sarkisyan, Dmitry A. Gorbachev, Evgeny S. Egorov, Alexander S. Mishin, Konstantin A. Lukyanov, Zbigniew Dauter, Sergei Pletnev

**Affiliations:** 1 Shemyakin-Ovchinnikov Institute of Bioorganic Chemistry, Russian Academy of Sciences, Moscow, Russian Federation; 2 Faculty of Biology, Moscow State University, Moscow, Russian Federation; 3 Nizhny Novgorod State Medical Academy, Nizhny Novgorod, Russian Federation; 4 Synchrotron Radiation Research Section, Macromolecular Crystallography Laboratory, National Cancer Institute, Argonne, Illinois, United States of America; 5 Leidos Biomedical Research, Inc., Basic Research Program, Argonne, Illinois, United States of America; Berlin Institute of Technology, GERMANY

## Abstract

Phototoxic fluorescent proteins represent a sparse group of genetically encoded photosensitizers that could be used for precise light-induced inactivation of target proteins, DNA damage, and cell killing. Only two such GFP-based fluorescent proteins (FPs), KillerRed and its monomeric variant SuperNova, were described up to date. Here, we present a crystallographic study of their two orange successors, dimeric KillerOrange and monomeric mKillerOrange, at 1.81 and 1.57 Å resolution, respectively. They are the first orange-emitting protein photosensitizers with a tryptophan-based chromophore (Gln65-Trp66-Gly67). Same as their red progenitors, both orange photosensitizers have a water-filled channel connecting the chromophore to the β-barrel exterior and enabling transport of ROS. In both proteins, Trp66 of the chromophore adopts an unusual *trans-cis* conformation stabilized by H-bond with the nearby Gln159. This *trans-cis* conformation along with the water channel was shown to be a key structural feature providing bright orange emission and phototoxicity of both examined orange photosensitizers.

## Introduction

Photosensitizers are the chromophores that generate reactive oxygen species (ROS) upon light irradiation. Until 2006, all known photosensitizers have been chemical compounds introduced into living systems exogenously. GFP-like red fluorescent protein KillerRed was the first genetically encoded photosensitizer that could be directly expressed by target cells [1]. Upon green or orange (530–590 nm) light irradiation, KillerRed generates ROS that damage the neighboring molecules. Only four such photosensitizers are known up to date: GFP-like proteins KillerRed [1] (λ_ex_/λ_em_ 585/610 nm) and its monomeric variant SuperNova [2] (λ_ex_/λ_em_ 579/610 nm), and FMN-binding proteins miniSOG (λ_ex_ 458 and 473 nm, λ_em_ 500 and 528 nm) [3] and Pp2FbFP L30M [4] (λ_ex_ 448 and 475 nm, λ_em_ 495 and 523 nm). Genetically encoded photosensitizers are a promising optogenetic tool for light-induced production of reactive oxygen species at desired locations within cells in vitro or whole body in vivo resulting in controlled elimination of specific cell populations, target protein inactivation, DNA damage, etc. [1, 2, 5–12]. Crystallographic studies showed that a unique structural feature observed in GFP-like photosensitizers is the water-filled channel extending along the β-barrel axis from the chromophore to the end of the barrel [2, 13, 14].

**Fig 1:**
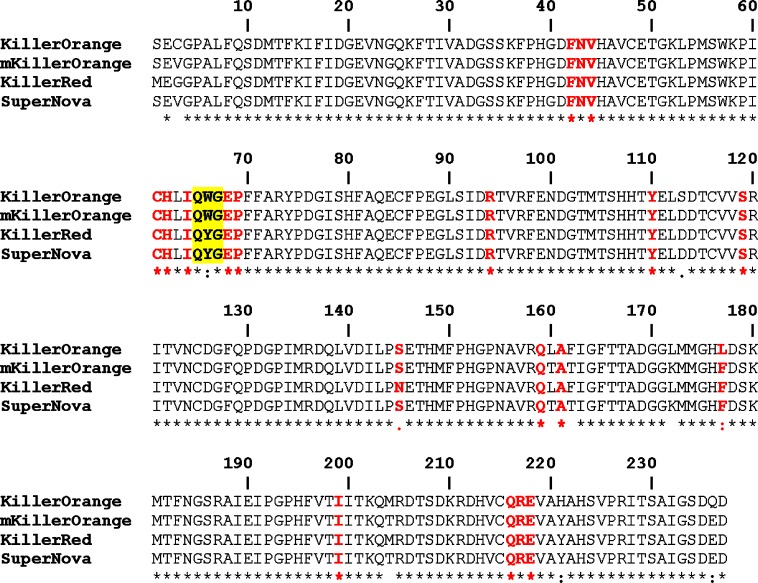
Alignment of the amino acid sequences of photosensitizers (overall identity ~95%). The residues in the chromophore nearest environment are shown in bold red and the chromophore forming triad is highlighted in yellow.

To further expand the toolkit of available phototoxic proteins we have very recently come up with a blue-shifted KillerRed variant carrying tryptophan-based chromophore (substitutions: Gly3Cys, Tyr66Trp, Asp113Ser, Asn145Ser, Phe177Leu, Tyr221His, Glu236Gln; Fig 1) named KillerOrange [15]. We also constructed monomeric mKillerOrange by introduction of the single Tyr66Trp substitution in SuperNova, a monomeric variant of KillerRed [2].

**Fig 2:**
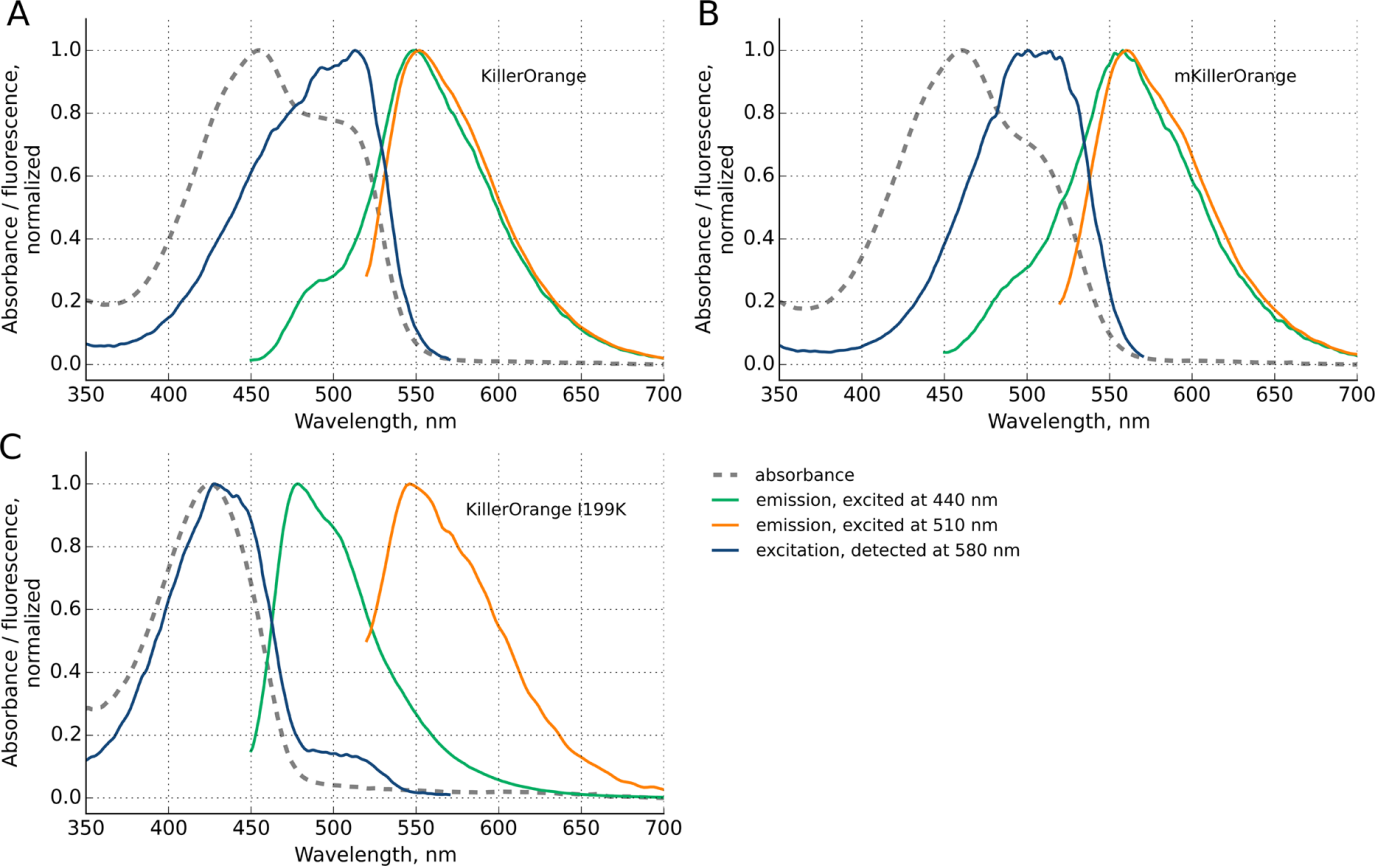
Normalized Spectra of (A) KillerOrange, (B) mKillerOrange, and (C) KillerOrange-L199K: Absorption (dashed grey lines), Excitation spectra for λ_em_ 580 nm (blue lines), and Emission spectra for λ_ex_ 440 nm (green lines) and 510 nm (orange lines).

The absorbance spectra of KillerOrange and mKillerOrange possess two overlapped bands with maxima at approximately 455 nm and 514 nm **(** Fig 2). Excitation at these wavelengths produces weak cyan (λ_em_ ~480 nm) and bright orange (λ_em_ ~555 nm) fluorescence, respectively. Most likely, the shorter and longer wavelength forms correspond to the CFP-like [16–18] and mHoneyDew-like [19] chromophores, respectively. Unlike parental KillerRed, which is toxic under green/orange light illumination, KillerOrange develops phototoxicity under blue/cyan light. The new orange variants expand the palette of genetically encoded photosensitizers and in combination with KillerRed they would make a useful pair for independent simultaneous control of two cell populations [15].

Here we present 1.81 and 1.57 Å crystal structures of two new orange-emitting photosensitizers with tryptophan-based chromophore (Gln65-Trp66-Gly67), dimeric KillerOrange and monomeric mKillerOrange (Fig 1) and discuss the results of the complementary mutagenesis experiments carried out to elucidate both the orange emission and the phototoxicity of these proteins.

## Materials and Methods

### Protein Expression, Purification, and Crystallization

The plasmids KillerOrange/pQE-30 and mKillerOrange/pQE-30 were transformed into *E*. *coli* XL1 Blue cells. The proteins were expressed in 3L of LB supplemented with 100 mg/L ampicillin by overnight incubation at 37°C. No induction by IPTG was applied since promoter leakage was sufficient for effective expression. Cells were resuspended in phosphate buffer (pH 7.4, PanEco, Russia), and lysed by sonication. Supernatant clarified by centrifugation was applied to a Talon metal-affinity resin (Clontech, USA) and washed with 10 column volumes of phosphate buffer saline (pH 7.4; 10 mM phosphate buffer, 137 mM NaCl, 2.7 mM KCl). The target protein was then eluted with the same phosphate buffer containing 250 mM of imidazole. Final purification was achieved by size-exclusion chromatography on a Superdex 200 HiLoad (16/60) (Amersham, USA). Protein concentration and buffer exchange were performed in 10 kDa molecular-weight cutoff concentration units (VivaScience, USA).

Crystals of KillerOrange and mKillerOrange were grown by hanging drop vapor diffusion method at 20°C. Each drop consisted of 2 μl of ~22 mg/ml protein solution in 20 mM Tris pH 8.0, 200 mМ NaCl, mixed with an equal amount of the respective reservoir solution. For KillerOrange, it contained 0.02M Na acetate pH 5.0, 1% (w/v) gamma-PGA (Na^+^ form, LM), 3% (w/v) PEG 4K; (where PGA-LM is poly-gamma-glutamic acid low molecular weight polymer). For mKillerORange, it contained 0.09M citric acid pH 3.5 22.5% PEG 3350. The crystals reached their final size in two weeks.

### X-ray Data collection, structure solution, and crystallographic refinement

Diffraction data were collected from a single crystal flash-cooled in a 100 K nitrogen stream. Prior to cooling, the crystal was transferred to a cryoprotecting solution containing 20% of glycerol and 80% of reservoir solution. The data were collected at a wavelength of 1 Å with a MAR300 CCD detector at the SER-CAT beamline 22ID (Advanced Photon Source, Argonne National Laboratory, Argonne, IL) and were processed with *HKL2000* [20].

Crystal structures of both phototoxic orange FPs were solved by the molecular replacement method with *MOLREP* [21, 22], using the coordinates of KillerRed monomer (PDB ID: 3GB3; [23]) as a search model. Structure refinement was performed with *REFMAC5* [24], alternating with manual revision of the model using *COOT* [25]. Crystallographic data and refinement statistics are given in Table 1. The coordinates and structure factors of the KillerOrange and mKillerOrange were deposited in the Protein Data Bank under accession codes 4ZFS and 4ZBL, respectively.

**Table 1:** Crystallographic data and refinement statistics.

Protein	KillerOrange (PDB_ID: 4ZFS)	mKillerOrange (PDB_ID: 4ZBL)
**Crystallographic data**		
Space group	C222_1_	P3_1_
Cell dimensions (Å)	a = 128.9 b = 202.1 c = 116.7	a = 64.2, b = 64.2, c = 47.4
*Z/(Z’)*	40 (5)	3 (1)
Estimated solvent content (%)	56	43
Temperature (K)	100	100
Wavelength (Å)	1.00	1.00
Resolution range (Å)	30.0–2.00 (2.07–2.00)*	30.0–1.57 (1.63–1.57)*
Total observations	1,724,142	115,789
Unique reflections observed	101,443	29,879
Redundancy	17.0	3.9 (3.5)*
*I/σ (I)*	20.5 (6.8)*	31.3 (4.9)*
*R*_*merge*_	0.124 (0.435)*	0.041(0.240)*
Completeness (%)	100.0 (100.0)*	98.1(96.0)*
**Refinement statistics**		
Non-H atoms in model		
Protein	9206	1,908
Water	862	158
Citrate	-	13
Glycerol	-	12
*R*_work_	0.192 (99.0%)**	0.144 (95.0%)**
*R*_free_	0.236 (1.0%)**	0.175 (5.0%)**
Mean B factor/(RMSD) (Å^2^)		
Protein atoms		
Main chain	42.9 (2.7)	13.9 (1.0)
Side chain	49.8 (3.8)	18.7 (2.2)
RMSD from ideal values:		
Bond lengths (Å)	0.019	0.023
Bond angles (°)	2.1	2.3
Torsion angles (period 3; °)	16	13
Chirality (Å^3^)	0.14	0.17
General planes (Å)	0.004	0.013
**Ramachandran statistics (%)**		
Preferred / Allowed / Outliers	95.2 / 3.4 / 1.4	95.2 / 3.2 / 1.6

*Values in parentheses are given for the highest-resolution shells.

**Percent of the data reserved for working and free sets.

### Mutagenesis and photophysical characterization

The KillerOrange mutant variants were obtained by site-directed mutagenesis using self-assembly cloning [26]. SuperNova-encoding plasmid was kindly provided by Prof. Takeharu Nagai. Synthetic DNA oligonucleotides for mutagenesis were purchased from Evrogen (Russia). PCRs were carried out using PTC-100 thermal cycler (MJ Research, USA). PCR products and products of digestion were purified by gel electrophoresis and extraction of DNA using Cleanup Standard Kit (Evrogen, Russia).

Mutants were cloned into a pQE-30 vector (Qiagen, USA). Small-scale expression was performed in *E*. *coli* XL1 Blue strain (Invitrogen, USA) grown at 37°C on Petri dishes with LB agar (100 mg/ml ampicillin), with no IPTG induction. After cell sonication, mutant proteins were purified using TALON metal affinity resin (Clontech, USA). Absorption and excitation–emission spectra of the purified proteins were recorded with Varian Cary 100 UV/VIS spectrophotometer and Varian Cary Eclipse fluorescence spectrophotometer, respectively. Quantum yield for orange emission of mutant proteins was determined by direct comparison with that of KillerOrange (assuming quantum yield of KillerOrange to be 0.42 [15]). The extinction coefficients were determined as following. To determine the concentration of mature (chromophore-containing) protein, aliquots of protein were denatured in 5M NaOH and their absorption spectra were measured. Under these conditions, proteins with tryptophan-based chromophores show an absorption peak at 463 nm with extinction coefficient of 46000 M^-1^cm^-1^ enabling determination of the protein concentration [15]. The extinction coefficient of the native protein was calculated from the absorption spectra of the aliquots of the same concentration in PBS pH 7.5.

The phototoxicity of KillerOrange and its mutants was evaluated by bacterial cell killing test described in [15]. Briefly, we mixed *E*. *coli* cells expressing EGFP with *E*. *coli* cells expressing the tested mutant, illuminated half of the suspension with a strong blue light, while keeping another half in the dark. We then plated each aliquot on Petri dishes, let the colonies grew overnight, washed the bacteria off from the plates, and analyzed the EGFP/KillerOrange mutant ratio in the suspension using fluorescence-activated cell sorting.

## Results

### Overall structure

The crystal asymmetric unit of KillerOrange contains two dimers and one monomer forming a dimer with the symmetry related subunit, while the crystal asymmetric unit of mKillerOrange contains one monomer. The dimeric structure of KillerOrange comprises two monomers related by a non-crystallographic 2-fold symmetry axis that are positioned at an angle of ~120° relative to each other. The contact area (~1400 Å^2^ per monomer) between the subunits is stabilized by symmetry related van der Waals contacts, two weak hydrogen bonds between the side chain of His148 (A/D) and the carbonyl of Glu146 (D/A), and two strong peripheral salt bridges between Glu99 (A/D) and Arg158 (D/A). Two aromatic side chains of Phe162 from each subunit make a stabilizing stack (at ~3.6 Å) shielded by side chains of four nearest His148/176 (A/D) and two Leu160 (D/A) residues at the interface center near the symmetry axis. In KillerOrange dimer, the irregular C-terminal tail (residues 224–230) sticks to the surface of the interacting subunits, contributing to the interface stabilization. The contact surface between the monomers of the adjacent dimers is smaller (~735 Å^2^ per monomer) than within a dimer and is stabilized by two salt bridges, two hydrogen bonds, and a few hydrophobic contacts. Similar to parental SuperNova [2], destabilization of central hydrophobic patch in the dimeric KillerOrange by Leu160Thr and Phe162Thr replacements ensures a monomeric state of mKillerOrange.

**Fig 3:**
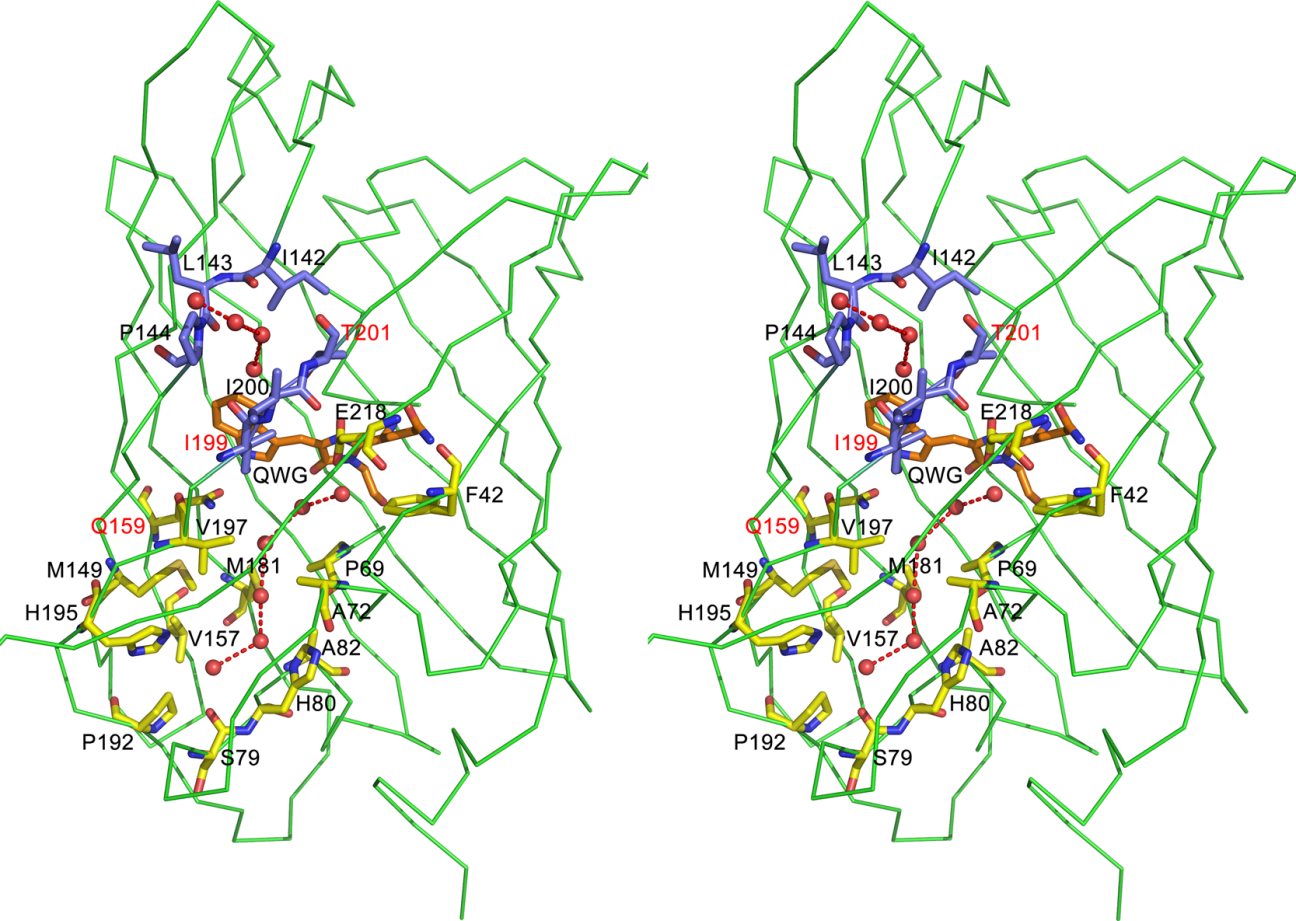
Stereoview of the water channel (residues shown in yellow) with a chain of seven water molecules (red spheres) and the pore (residues shown in blue) filled with four water molecules. Residues mutated in this work are labeled in red. (Figure created with *PYMOL* [37])

The principal structural fold of the monomer in KillerOrange/mKillerOrange, shared with all members of the GFP family, is an 11-stranded β-barrel having loops from both sides and the chromophore (matured from Gln65-Trp66-Gly67 sequence) embedded in the middle of the internal α-helix wound around the β-barrel axis. The structure shows existence of a pore formed by the backbone of Ile142, Leu143, Pro144, Ile199, Ile200, and Thr201. The pore is filled by four water molecules connecting the indole moiety of the chromophore with the protein exterior. (Fig 3). The pore was found in the earlier reported photosensitizers KillerRed and SuperNova [2, 13, 14] and in numerous non-phototoxic fluorescent proteins, such as TurboGFP (variant of ppluGFP2, *Pontellina plumata*) [27]; wild type zGFP506, zYFP538, and zRFP574 (*Zoanthus*) [28, 29]; and eqFP578 (*Entacmaea quadricolor*) [30]. The authors of [27] suggested that the pore affords an additional oxygen supply to the chromophore and accelerates its maturation.

Similar to their red progenitors, KillerOrange and mKillerOrange, show the presence of a water-filled channel extending along the β-barrel axis (Fig 3). A continuous chain of seven hydrogen-bonded water molecules in the channel covers the distance of ~20 Å from catalytic Glu218 in the chromophore area to Pro192 at the bottom of β-barrel. Each water molecule forms hydrogen bonds with the preceding and following water molecules and with the proximal amino acid residues lining the channel. This water-filled channel is a distinct feature of GFP-like photosensitizers and was suggested to be a key structural element providing their phototoxicity [13,14]. In the channel of mKillerOrange, we found a glycerol molecule, presumably coming from the cryoprotecting solution and replacing three out of seven water molecules present in KillerOrange.

### Chromophore area

**Fig 4:**
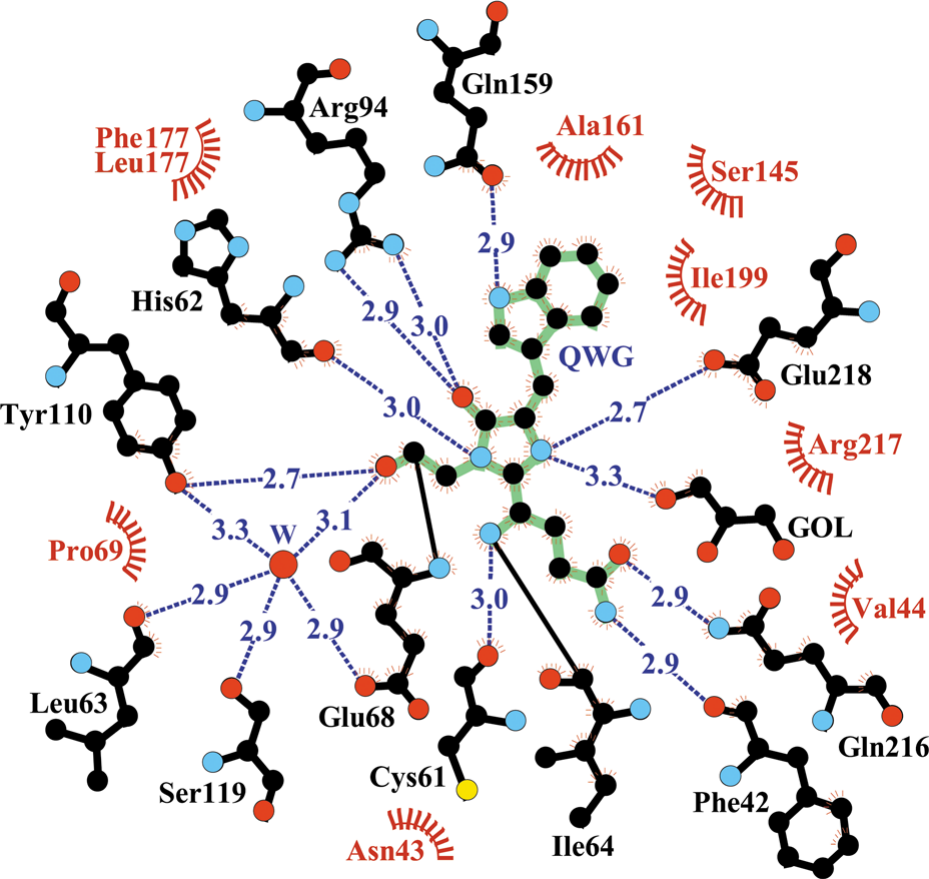
The nearest chromophore environment of KillerOrange/mKillerOrange. The crystal structures of two proteins have a difference in position 177 –(Leu and Phe, respectively) and in the presence of glycerol (GOL—component of the cryo-protecting solution) in mKillerOrange structure. Hydrogen bonds (≤3.3 Å) are shown as blue dashed lines, water molecules (W) as red spheres, and van der Waals contacts (≤3.9 Å) as red “eyelashes”. (Figure prepared with *LIGPLOT/HBPLUS*, [38]).

**Fig 5:**
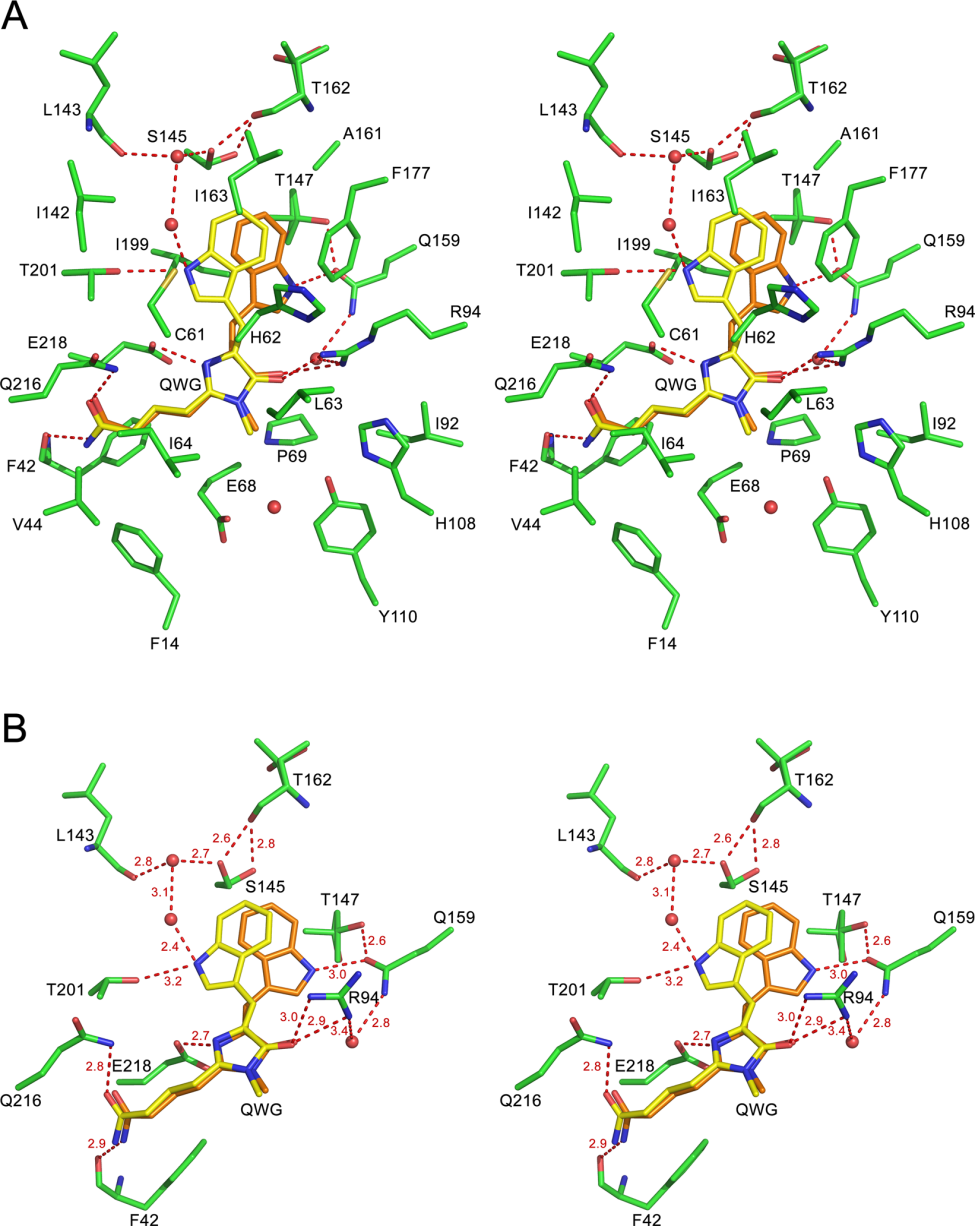
Stereoview of the nearest chromophore environment of mKillerOrange. *Trans-cis* (~85%) and *cis-cis* (~15%) conformations of the chromophore are shown in orange and yellow, respectively. (A) A complete set of residues surrounding the chromophore. (B) Residues forming H-bond network around the chromophore.

**Fig 6:**
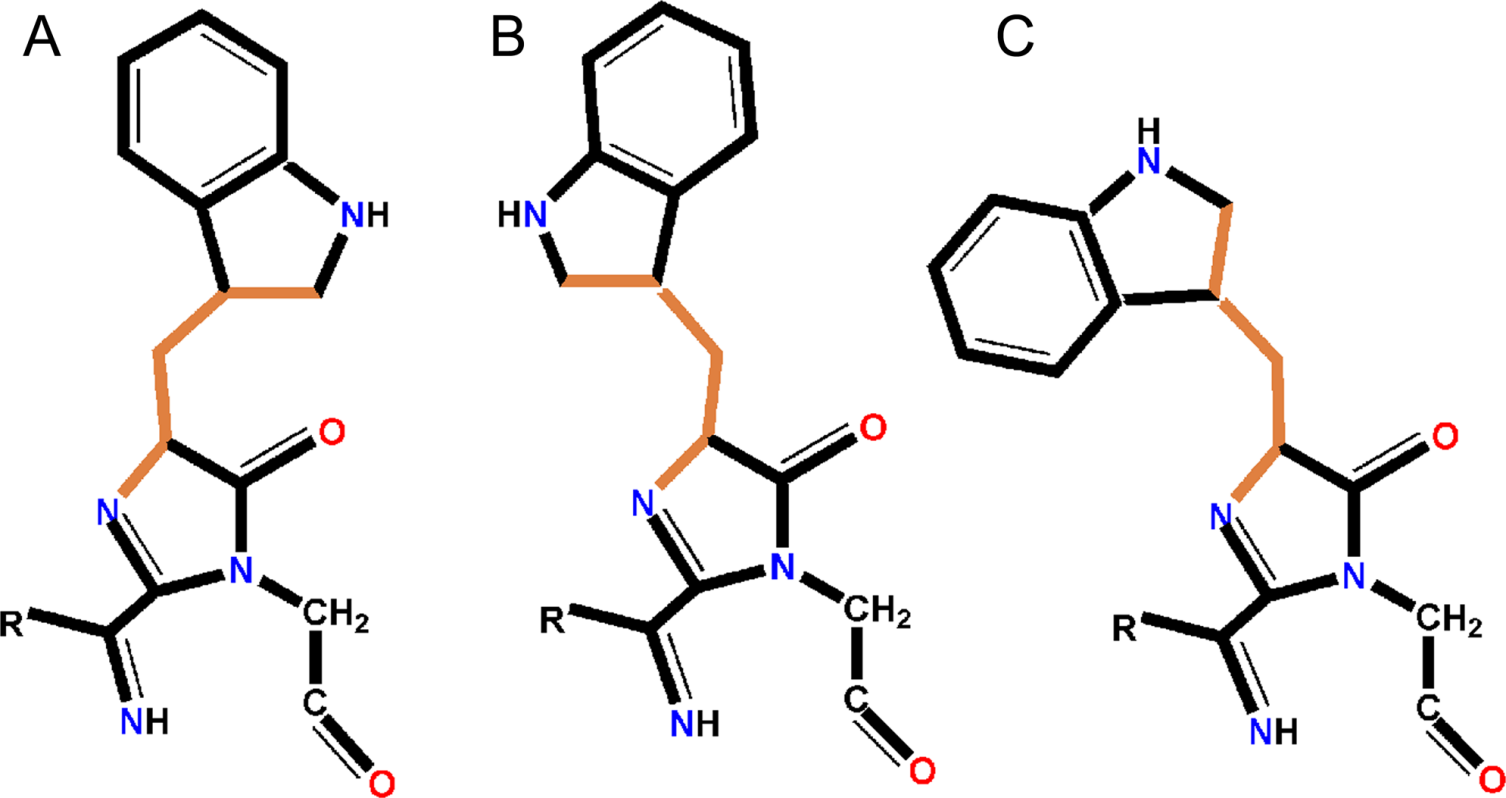
Schematic representation of the chromophore. Trp66 isomers described by χ1 and χ2 torsional angles around C^α^-C^β^ and C^β^-C^γ^ bonds, respectively (the corresponding dihedral angles are defined by atoms N-C^α^-C^β^- C^γ^ and C^α^-C^β^-C^γ^-C^δ^; shown in orange). (A) *trans-cis* (KillerOrange and mKillerOrange structures; present work), (B) *cis-cis* (Cerulean structure at pH 7; [18]), (C) *cis-trans* (Cerulean structure at pH 5; [17]). (Figure was created with ChemDraw [39]).

Posttranslational modification of the chromophore-forming sequence Gln65-Trp66-Gly67 (QWG) in KillerOrange/mKillerOrange results in a chromophore consisting of a five-membered imidazolinone heterocycle and an aromatic nine-membered indole ring. In both proteins, the nearest environment of the QWG chromophore (within 3.9 Å) is nearly identical and consists of 19 residues with the only difference at position 177 occupied by Leu and Phe in KillerOrange and mKillerOrange, respectively (Figs 1, 4 and 5). The chromophore forms nine direct hydrogen bonds with its immediate environment, including those with the catalytic Glu218 and Arg94. The side chain of Trp66 of the chromophore in KillerOrange and mKillerOrange adopts an unusual *trans-cis* conformation, not seen for other Trp-based chromophores before. It is described by the χ1/χ2 torsion angles of 175°/16° and -178°/26°, respectively (rotation around C^α^-C^β^ and C^β^-C^γ^ bonds defined by dihedral angles N-C^α^-C^β^- C^γ^ and C^α^-C^β^-C^γ^-C^δ^) (Table 2, Fig 6). A higher resolution structure of mKillerOrange also shows the traces (~10–15%) of alternative *cis-cis* conformation with χ1/χ2 of -5°/-8°. In both structures, the indole ring of Trp66 in a dominant *trans-cis* conformation is stabilized by hydrogen bond between the indole nitrogen and the side chain of Gln159 and shows a noticeable deviation from the chromophore planarity (χ2 ~16–26°). Conversely, in the minor *cis-cis* conformation the indole ring is nearly coplanar with the chromophore imidazolinone ring and is stabilized by a hydrogen bond with Thr201.

**Table 2:** Conformational states of Trp side chain in the Trp based chromophores ^a^.

KillerOrange		mKillerOrange		mKillerOrange		Cerulean (pH 7)^c^		Cerulean (pH 5)^d^	
*trans-cis*		*trans-cis* (85%)		*cis-cis* (15%)		*cis-cis*		*cis-trans*	
χ1^b^	χ2^b^	χ1	χ2	χ1	χ2	χ1	χ2	χ1	χ2
175	16	-178	26	5	-8	5	-6	-1	180

^a^ Conformation of the *trans-trans* type is sterically prohibited.

^b^ The χ1 andχ2 torsion angles (grad) around C^α^-C^β^ and C^β^-C^γ^ bonds (defined by dihedral angles presented by atoms N-C^α^-C^β^- C^γ^ and C^α^-C^β^-C^γ^-C^δ^, respectively).

^c^[18].

^d^[17].

### Structure-based site-directed mutagenesis

Based on the obtained crystal structures we designed site-directed mutagenesis experiments aimed at identifying of the residues responsible for phototoxicity of KillerOrange and mKillerOrange. Gln159 and Thr201 were identified as sites affecting the equilibrium of *trans-cis* and *cis-cis* conformations of Trp66. Also, in the earlier study of KillerRed [14], Glu68 located right after the chromophore was shown to influence the protein phototoxicity. To test these chromophore-adjacent residues, we have prepared the mutants with the following single substitutions: Gln159Gly or Glu68Gln in KillerOrange, and Thr201Ala in mKillerOrange. Unfortunately, all three mutations resulted in almost immature proteins that impeded any conclusion regarding the specific role these residues play in (m)KillerOrange phototoxicity and fluorescence.

**Fig 7:**
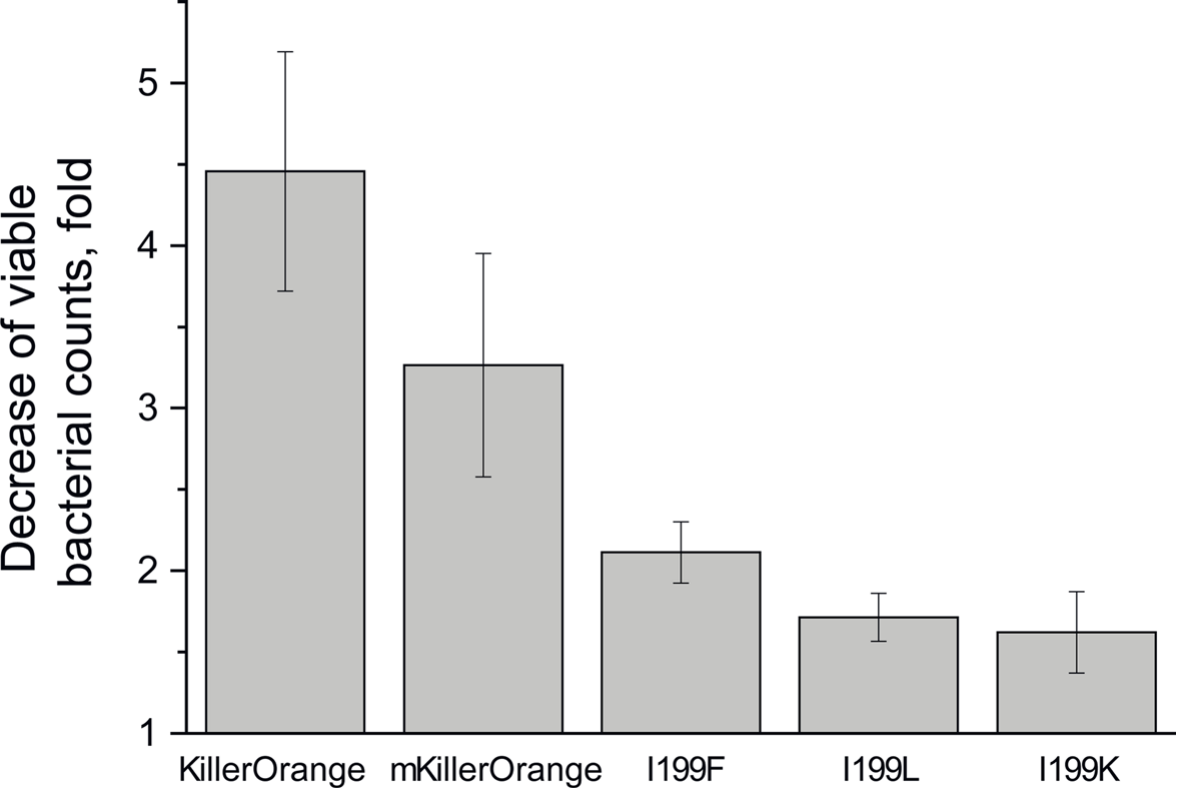
Relative phototoxicity of KillerOrange variants. Histogram shows light-induced decrease in viable bacterial counts of *E*. *coli* expressing the designated proteins. Bacterial suspensions were illuminated with 460/20 nm LED (320 mW) for 400 s.

We have also applied a site-directed mutagenesis to block the water-filled channel claimed to be crucial for ROS generation [13, 14]. Ile199Phe and Ile199Leu replacements resulted in a considerable loss of phototoxicity (Fig 7), while the spectral properties of the corresponding mutants were rather similar to those of KillerOrange (Table 3). Surprisingly, replacement of Ile199 with a positively charged bulky Lys resulted in a low-phototoxic protein with predominantly cyan emission (Fig 2C, Table 3). The other “water channel” mutants Ala82Ser and Ile199Met/Arg were dim and exhibited a very low maturation rates, preventing direct comparison of their phototoxicity with that of the parental KillerOrange.

**Table 3:** Spectral characteristics of KillerOrange and its mutants.

	KillerOrange	mKillerOrange	I199F	I199L	I199K
Absorption max, nm (EC^a^)	452 (35000)	458 (35000)	452 (29000)	450 (42000)	427 (43000)
EC at 514 nm	19000	20000	8000	23000	600
Cyan emission max, nm	~480 (shoulder)	~480 (shoulder)	~480 (shoulder)	~480 (shoulder)	478 (main peak)
Orange emission max, nm	555 (major)	560 (major)	563 (major)	555 (major)	545 (minor)
Quantum yield, excitation at 510 nm	0.42	0.50	0.75	0.87	0.85

^a^ Extinction coefficient, M^-1^cm^-1^.

## Discussion

The phototoxicity of the GFP-like proteins is usually very low—the only known GFP-like photosensitizers with substantial phototoxicity reported to date are red-emitting KillerRed and its monomeric variant SuperNova with tyrosine-based chromophore. Replacement of the chromophore Tyr66 in KillerRed with Trp, despite a significant change of spectral properties, had little effect on phototoxicity of its (m)KillerOrange successors. Chromophores in red and orange photosensitizers have a nearly identical nearest amino acid environment with a similar interaction pattern showing the major difference in the area of the aromatic side chains of the chromophores.

In red photosensitizers, *cis* conformation of Tyr66 is stabilized by direct and water-mediated hydrogen bonds with Asn145 and Thr201, respectively. In orange photosensitizers, Trp66 adopts a dominant *trans-cis* and a minor *cis-cis* conformations stabilized by hydrogen bond with Gln159 and Thr201, respectively. The *trans-cis* isomer of (m)KillerOrange chromophore is the first example of such conformation among the known Trp-based chromophores. All earlier reported Trp-based chromophores adopted either *cis-cis* or *cis-trans* conformation and exhibited either cyan or green emission in case of protonated [17, 18] and anionic Trp66 [31], respectively (Fig 6, Table 2). Ile199Lys replacement in KillerOrange resulted in ~25 nm blue shift of absorption band maxima accompanied by significant decrease of the protein phototoxicity and raise of cyan emission (Figs 2 and 7, Table 3), presumably due to isomerization of Trp66 from dominant *trans-cis* to minor *cis-cis* conformation. We suggest that *trans-cis* conformation of the chromophore Trp66 in KillerOrange is a key structural feature for appearance of bright orange emission and phototoxicity.

The water-filled channel connecting the chromophore with exterior of the β-barrel was identified as a prerequisite feature for phototoxicity of GFP-based photosensitizers. The continuous chain of seven hydrogen bonded water molecules extending along the channel axis has been suggested to transport oxygen and ROS between the solvent and the chromophore [2, 13, 14, 32–34]. Alternatively, highly ordered water molecules could play a role of “electron wire”, conducting electrons from the external redox species, which was a subject of a number of publications, e.g., [35, 36].

The results of mutagenesis demonstrated that interruption of the string of hydrogen-bonded water molecules in the channel by introduction of bulky residues, Ile199Phe and Ile199Leu, results in decreased phototoxicity of the mutants, presumably, due to decelerated ROS production. All tested mutants I199Phe/Leu/Lys possessed remarkably higher quantum yield of orange fluorescence compared to the parental protein (Table 3), suggesting that bulky residues at position 199 could restrict the freedom of the chromophore movements suppressing its non-radiative transition.

## Conclusions

Here, we presented the X-ray structures of dimeric KillerOrange and monomeric mKillerOrange, the only known to date GFP-photosensitizers with Trp-based chromophore. Same as for its red progenitors, KillerRed and SuperNova, to exhibit phototoxicity KillerOrange and its monomer require the presence of the water-filled channel connecting the chromophore to the β-barrel exterior and enabling transport of ROS. Even a partial blockage of the channel decelerates production of ROS reducing the protein phototoxicity. Specifically for KillerOrange and its monomer, never reported before dominant *trans-cis* conformation of the chromophore Trp66, stabilized by H-bond with the adjacent Gln159, provides orange fluorescence of both photosensitizers and enables their phototoxicity. The third feature, worth mentioning in conclusion, is the position #199 occupied by Ile. Replacement of Ile199 by bulky Phe, Leu or Lys decreases phototoxicity and results in almost doubled quantum yields of 0.75–0.87. The structural and mutagenesis data presented here provide a solid base for further investigation of the mechanisms of ROS generation and for development of new, more efficient genetically encoded photosensitizers.

## References

[1] Bulina ME, Chudakov DM, Britanova OV, Yanushevich YG, Staroverov DB, Chepurnykh TV, et al A genetically encoded photosensitizer. Nat Biotechnol. 2006 24: 95–99. 16369538 10.1038/nbt1175

[2] Takemoto K, Matsuda T, Sakai N, Fu D, Noda M, Uchiyama S, et al SuperNova, a monomeric photosensitizing fluorescent protein for chromophore-assisted light inactivation. 2013 Sci Rep. 3: 2629 10.1038/srep02629 24043132 PMC3775092

[3] Shu X, Lev-Ram V, Deerinck TJ, Qi Y, Ramko EB, Davidson MW, et al A genetically encoded tag for correlated light and electron microscopy of intact cells, tissues, and organisms. PLoS Biol. 2011 9: e1001041 10.1371/journal.pbio.1001041 21483721 PMC3071375

[4] Torra J, Burgos-Caminal A, Endres S, Wingen M, Drepper T, Gensch T, et al Singlet oxygen photosensitisation by the fluorescent protein Pp2FbFP L30M, a novel derivative of Pseudomonas putida flavin-binding Pp2FbFP. Photochem Photobiol Sci. 2015 14: 280–287. 10.1039/c4pp00338a 25375892

[5] Serebrovskaya EO, Gorodnicheva TV, Ermakova GV, Solovieva EA, Sharonov GV, Zagaynova EV, et al Light-induced blockage of cell division with a chromatintargeted phototoxic fluorescent protein. Biochem J. 2011 435: 65–71. 10.1042/BJ20101217 21214518

[6] Ivashchenko O, Van Veldhoven PP, Brees C, Ho YS, Terlecky SR, Fransen M. Intraperoxisomal redox balance in mammalian cells: oxidative stress and interorganellar crosstalk. Mol Biol Cell. 2011 22: 1440–1451. 10.1091/mbc.E10-11-0919 21372177 PMC3084667

[7] Williams DC, Bejjani RE, Ramirez PM, Coakley S, Kim SA, Lee H, et al Rapid and permanent neuronal inactivation in vivo via subcellular generation of reactive oxygen with the use of KillerRed. Cell Rep. 2013 5: 553–563. 10.1016/j.celrep.2013.09.023 24209746 PMC3877846

[8] Kuznetsova DS, Shirmanova MV, Dudenkova VV, Subochev PV, Turchin IV, Zagaynova EV, et al Photobleaching and phototoxicity of KillerRed in tumor spheroids induced by continuous wave and pulsed laser illumination. J Biophotonics. 2015 10.1002/jbio.201400130 25648724

[9] Lin JY, Sann SB, Zhou K, Nabavi S, Proulx CD, Malinow R, et al Optogenetic inhibition of synaptic release with chromophore-assisted light inactivation (CALI). Neuron. 2013 79: 241–253. 10.1016/j.neuron.2013.05.022 23889931 PMC3804158

[10] Ryumina AP, Serebrovskaya EO, Shirmanova MV, Snopova LB, Kuznetsova MM, Turchin IV, et al Flavoprotein miniSOG as a genetically encoded photosensitizer for cancer cells. Biochim Biophys Acta. 2013 1830: 5059–5067. 10.1016/j.bbagen.2013.07.015 23876295

[11] Lukyanov KA, Serebrovskaya EO, Lukyanov S, Chudakov DM. Fluorescent proteins as light-inducible photochemical partners. Photochem Photobiol Sci. 2010 9: 1301–1306. 10.1039/c0pp00114g 20672171

[12] Sano Y, Watanabe W, Matsunaga S. Chromophore-assisted laser inactivation–towards a spatiotemporal-functional analysis of proteins, and the ablation of chromatin, organelle and cell function. J Cell Sci. 2014 127: 1621–1629. 10.1242/jcs.144527 24737873

[13] Carpentier P, Violot S, Blanchoin L, Bourgeois D. Structural basis for the phototoxicity of the fluorescent protein KillerRed. FEBS Lett. 2009 583: 2839–2842. 10.1016/j.febslet.2009.07.041 19646983

[14] Pletnev S, Gurskaya NG, Pletneva NV, Lukyanov KA, Chudakov DM, Martynov VI, et al Structural basis for phototoxicity of the genetically encoded photosensitizer KillerRed. J Biol Chem. 2009 284: 32028–32039. 10.1074/jbc.M109.054973 19737938 PMC2797274

[15] Sarkisyan KS, Zlobovskaya OA, Gorbachev DA, Bozhanova NG, Sharonov GV, Egorov ES, et al KillerOrange, a genetically encoded photosensitizer activated by blue light. PLoS One. Forthcoming 2015. 10.1371/journal.pone.0145287 PMC4683004 26679300

[16] Bae JH, Rubini M, Jung G, Wiegand G, Seifert MH, Azim MK, et al Expansion of the genetic code enables design of a novel "gold" class of green fluorescent proteins. J Mol Biol. 2003 328: 1071–1081. 12729742 10.1016/s0022-2836(03)00364-4

[17] Malo GD, Pouwels LJ, Wang M, Weichsel A, Montfort WR, Rizzo MA, et al X-ray structure of Cerulean GFP: a tryptophan-based chromophore useful for fluorescence lifetime imaging. Biochemistry. 2007 46: 9865–9873. 17685554 10.1021/bi602664c

[18] Lelimousin M, Noirclerc-Savoye M, Lazareno-Saez C, Paetzold B, Le VS, Chazal R, et al Intrinsic dynamics in ECFP and Cerulean control fluorescence quantum yield. Biochemistry. 2009 48: 10038–10046. 10.1021/bi901093w 19754158

[19] Shaner NC, Campbell RE, Steinbach PA, Giepmans BN, Palmer AE, Tsien RY. Improved monomeric red, orange and yellow fluorescent proteins derived from Discosoma sp. red fluorescent protein. Nat Biotechnol. 2004 22: 1567–1572. 15558047 10.1038/nbt1037

[20] Otwinowski Z, Minor W. Processing of X-ray diffraction data collected in oscillation mode. Methods Enzymol. 1997 276: 307–326. 10.1016/S0076-6879(97)76066-X 27754618

[21] Winn MD, Ballard CC, Cowtan KD, Dodson EJ, Emsley P, Evans PR, et al Overview of the CCP4 suite and current developments. Acta Crystallogr D Biol Crystallogr. 2011 67: 235–242. 10.1107/S0907444910045749 21460441 PMC3069738

[22] Vagin A, Teplyakov A. Molecular replacement with MOLREP. Acta Crystallogr D Biol Crystallogr. 2010 66: 22–25. 10.1107/S0907444909042589 20057045

[23] Yang F, Moss LG, Phillips GN Jr. The molecular structure of green fluorescent protein. Nat Biotechnol. 1996 14: 1246–1251. 9631087 10.1038/nbt1096-1246

[24] Murshudov GN, Vagin AA, Dodson EJ. Refinement of macromolecular structures by the maximum-likelihood method. Acta Crystallogr D Biol Crystallogr. 1997 53: 240–255. 15299926 10.1107/S0907444996012255

[25] Emsley P, Cowtan K. Coot: model-building tools for molecular graphics. Acta Crystallogr D Biol Crystallogr. 2004 60: 2126–2132. 15572765 10.1107/S0907444904019158

[26] Matsumoto A, Itoh TQ. Self-assembly cloning: a rapid construction method for recombinant molecules from multiple fragments. Biotechniques. 2011 51: 55–56. 10.2144/000113705 21781054

[27] Evdokimov AG, Pokross ME, Egorov NS, Zaraisky AG, Yampolsky IV, Merzlyak EM, et al Structural basis for the fast maturation of Arthropoda green fluorescent protein. EMBO. 2006 Rep 7: 1006–1012. 10.1038/sj.embor.7400787 PMC1618374 16936637

[28] Pletneva N, Pletnev V, Tikhonova T, Pakhomov AA, Popov V, Martynov VI, et al Refined crystal structures of red and green fluorescent proteins from the button polyp Zoanthus. Acta Crystallogr D Biol Crystallogr. 2007 63: 1082–1093. 17881826 10.1107/S0907444907042461

[29] Pletneva NV, Pletnev SV, Chudakov DM, Tikhonova TV, Popov VO, Martynov VI, et al Three-dimensional structure of yellow fluorescent protein zYFP538 from Zoanthus sp. at the resolution 1.8 angstrom. Bioorg Khim. 2007 33: 421–430. 17886433 10.1134/s1068162007040048

[30] Pletneva NV, Pletnev VZ, Shemiakina II, Chudakov DM, Artemyev I, Wlodawer A, et al Crystallographic study of red fluorescent protein eqFP578 and its far-red variant Katushka reveals opposite pH-induced isomerization of chromophore. Prot Sci. 2011 20 (7): 1265–1274. 10.1002/pro.654 PMC3149199 21563226

[31] Pletnev VZ, Pletneva NV, Sarkisyan KS, Mishin AS, Lukyanov KA, Goryacheva EA, et al Crystal structure of green fluorescent protein NowGFP with an anionic tryptophan-based chromophore. Acta Crystallogr D Biol Crystallogr. 2015 D71: 1699–1707. 10.1107/S1399004715010159 PMC4528802 26249350

[32] Vegh RB, Solntsev KM, Kuimova MK, Cho S, Liang Y, Loo BL, et al Reactive oxygen species in photochemistry of the red fluorescent protein "Killer Red". Chem Commun (Camb). 2011 47: 4887–4889. 21359336 10.1039/c0cc05713d

[33] Roy A, Carpentier P, Bourgeois D, Field M. Diffusion pathways of oxygen species in the phototoxic fluorescent protein KillerRed. Photochem Photobiol Sci. 2010 9: 1342–1350. 10.1039/c0pp00141d 20820672

[34] Vegh RB, Bravaya KB, Bloch DA, Bommarius AS, Tolbert LM, Verkhovsky M, et al Chromophore photoreduction in red fluorescent proteins is responsible for bleaching and phototoxicity. J Phys Chem B. 2014 118: 4527–4534. 10.1021/jp500919a 24712386 PMC4010289

[35] Lin J, Balabin IA, Beratan DN. The nature of aqueous tunneling pathways between electron-transfer proteins. Science. 2005 310: 1311–1313. 16311331 10.1126/science.1118316 PMC3613566

[36] Miyashita O, Okamura MY, Onuchic JN. Interprotein electron transfer from cytochrome c2 to photosynthetic reaction center: tunneling across an aqueous interface. Proc Natl Acad Sci U S A. 2005 102: 3558–3563. 15738426 10.1073/pnas.0409600102 PMC553326

[37] DeLano WL. The PyMOL Molecular Graphics System. 2002 http://www.pymol.org.

[38] Wallace AC, Laskowski RA, Thornton JM. LIGPLOT: a program to generate schematic diagrams of protein-ligand interactions. Protein Eng. 1995 8: 127–134. 7630882 10.1093/protein/8.2.127

[39] Cambridge Soft Corporation 2003 http://www/camsoft.com.

